# Editorial: Novel imaging technologies for neuroinflammation

**DOI:** 10.3389/fneur.2022.1125045

**Published:** 2023-01-06

**Authors:** Yutong Liu, Weiguo Li

**Affiliations:** ^1^Department of Radiology, University of Nebraska Medical Center, Omaha, NE, United States; ^2^Department of Pharmacology and Experimental Neuroscience, University of Nebraska Medical Center, Omaha, NE, United States; ^3^Department of Radiology, Northwestern University, Chicago, IL, United States; ^4^Department of Biomedical Engineering, University of Illinois at Chicago, Chicago, IL, United States

**Keywords:** imaging, neuroinflammation, neurological diseases, neuroimmune dysfunction, MRI, PET, neurological disorders, neuroimmune system

This Research Topic focuses on imaging technologies for neuroinflammation, which is the underlying pathology of neurologic manifestations. As such, imaging plays an integral role in the diagnosis of neurological diseases, monitoring of disease progression, assessments of therapeutic efficacy, and treatment planning. The four papers (two research papers, one review, and one study protocol) in this Research Topic reflect the development and applications of neuroimaging for these areas of interest. One research paper used diffusion tensor imaging (DTI) to detect microstructural changes of the vestibulocochlear nerve in patients with Menière's disease (MD). The other research paper developed a semi-automated segmentation method to measure perivascular spaces in the CNS of patients showing neurologic impairments associated with COVID-19. The review paper offers a thorough literature review of MRI brainstem studies among Myalgic Encephalomyelitis/Chronic Fatigue Syndrome (ME/CFS) patients. The study protocol presented a study design that combines neuroimaging and fluid-based biomarker measurements aims to investigate the early interplay of beta-amyloid (Ab) deposition, neuroinflammation, synaptic dysfunction, and synaptic loss through observation of cognitively typical volunteers with three different levels of genetic (APOE-related) risk for late-onset Alzheimer's disease (AD).

Recent studies indicate that neuroinflammation may be related to tinnitus, hearing loss, and the onset of Menière's disease (1, 2). Neuroinflammatory conditions can lead to brain-blood barrier (BBB) permeability alterations and thus a change in the interstitial space composition, which in turn determines changes of water diffusion detected by DTI. As an advanced functional MRI technique, DTI explores microstructural integrity of nervous tissue using multi-directional diffusion-sensitizing gradients. Metrics calculated from DTI models include fractional anisotropy (FA), and apparent diffusion coefficient (ADC) that might serve as biomarkers for nerve fiber integrity. Microstructure changes caused by chronic inflammatory phenomena could be probed by subtle diffusion metrics alterations. In the paper by Yuan et al., DTI was applied on 13 patients with Menière's disease (MD) and 13 healthy controls to evaluate the microstructural changes of the vestibulocochlear nerve. The results showed a significant decrease in FA and an increase in ADC of the vestibulocochlear nerve in the MD patients compared with healthy controls and FA were negatively correlated with the dizziness handicap inventory scores. The decreased FA and increased ADC may indicate microstructural changes occurred in vestibulocochlear nerves, which may be edema, inflammation, the damage of neural fiber integrity, myelin, or axonal integrity in the active MD patients. However, while this study proved the feasibility of DTI in accessing vestibulocochlear nerve in the MD patients, it is necessary to perform further validation studies that include large patient population and neuropathological diagnosis from multiple centers. In addition, DTI study could combine with magnetic resonance spectroscopy as well as other techniques such as dynamic contrast enhancement (DCE) MRI and PET/MRI for longitudinal assessing the complicated processes and interactions during neuroinflammatory changes.

The paper by Langan et al. describes a semi-automated segmentation method to measure perivascular spaces (PVS) in COVID-19 patients on images acquired on a 7 Tesla scanner. This method uses a Frangi-based algorithm, producing automated data that were validated through comparison with manual results. While this was only a preliminary study and used a small sample size of COVID-19 patients (*n* = 10), it offers interesting results. Notable observations include significantly increased PVS presence, enlarged white matter volume in COVID-19 patients, significant correlations between PVS count and BMI, and between white matter volume and BMI within the COVID-19 patient cohort and within all subjects. These findings suggest that the proposed PVS semi-automated segmentation (PVSSAS) method provides an imaging biomarker of disease-associated neuroinflammation, as the increased presence of PVS indicates blood-brain barrier impairment. This impairment may impact or precede cascades leading to neuroinflammatory processes (3, 4). This study is immediately relevant as there are numerous reports of COVD-19 patients developing serious neurologic complications post-recovery, including fatigue, headache, anosmia, anxiety, and brain fog. There is plausible evidence suggesting that inflammatory processes underly these complications (5). As such, a non-invasive imaging biomarker of COVID-associated neuroinflammation is a powerful tool for the diagnosis and monitoring of neurologic complications in COVID-19 recovery. Interestingly, because patients were not scanned before infection, the authors suggested that it is difficult to determine whether increased PVS count is a *consequence* of COVID-19, or a *risk factor* of neurologic disorders following infection. Further study is clearly warranted, as it is critical for the prevention and treatment of COVID-associated neurologic disorders. The authors also noted other limitations of the study, such as the lack of a scoring system to evaluate the severity of the neurologic symptoms and the limited access to relevant patients records (e.g., plasma inflammatory markers). We believe it is imperative to test the proposed method on image data acquired on 1.5 and 3 Tesla scanners, as they are the mainstream clinical MRI field strengths. If successful, the proposed method should see increased clinical use.

The paper by Nelson et al. reviewed 11 MRI studies on brainstem in patients of Myalgic Encephalomyelitis/Chronic Fatigue Syndrome (ME/CFS). Of these studies, 10 investigated structural brain changes, three measured brain functional connectivity, and one performed diffusion tensor imaging (DTI). These studies demonstrated reduced white matter volume, impaired myelination, reduced conduction, abnormal functional connectivity between the brainstem and other brain regions, and brain changes to compensate brainstem dysfunctions. I want to mention that Figure 3 in the paper shows a nice summary of the literature review, and linked the symptoms of ME/CFS patients with underlying brainstem dysfunctions based on MRI findings. Despite numerous studies suggesting that the brainstem is involved in ME/CFS (6, 7), MRI study of the brainstem in ME/CFS patients only started very recently; this is shown in Figure 2. Notably, most imaging studies only measured structural changes in the brainstem. Only a few functional MRI studies were performed to measure functional connectivity, and only one study used DTI. This review demonstrated the merits of brainstem MRI in the research of ME/CFS, and highlighted the imperative to advanced MRI techniques such as fMRI and DTI to reveal functional and structural connectivity because brainstem is the crucial passage of nerves in both afferent and efferent directions.

Neuroinflammation plays a critical role in the pathogenesis of Alzheimer's disease (AD), the most common form of dementia worldwide (8). Knowledge of early pathophysiological changes in AD can provide benefit for preventive or potential therapeutic strategies for this non-reversible neurological disorder. In this issue, Snellman et al. reported the study protocol and basic characteristics of their ASIC-E4 study (“Beta-Amyloid, Synaptic loss, Inflammation and Cognition in healthy APOE +4 carriers”). This project combined neuroimaging techniques (PET and MRI) and fluid-based biomarker measurements aimed to study the early interplay of three key pathological features of AD, i.e., beta-amyloid (Ab) deposition, neuroinflammation and synaptic dysfunction and loss. The study enrolled 63 cognitively normal volunteers with three different levels of Apolipoprotein E-related risk for late-onset of AD. The baseline study has finished three PET scans with tracers targeting against Ab deposition (11C-PIB), activated glia (11C-PK11195) and synaptic vesicle glycoprotein 2A (11C-UCB-J), MRI including anatomical scans (T1-, T2-weighted, FLAIR/VISTA) and functional scanning (resting state-fMRI and DTI), cognitive testing, and blood testing and/or cerebrospinal fluid samples testing (on a subset of participants). Neuropsychological evaluation and blood biomarker measurements will be repeated after a 4-year follow-up period to evaluate the predictive value of the early neuroimaging findings. The ASIC-E4 project recruited rare homozygotic carriers of the APOE ε4, the major genetic risk factor for AD and studied them with the multimodal and multi-tracer approaches. In this regard, this study will bridge the gap related to limited knowledge of the synaptic and inflammatory changes and their interactions in individuals at risk of developing AD. Furthermore, this study could help identify the potential novel imaging markers and provide additional information about effect of APOE ε4. *In vivo* characterization of the biomarker profiles will, as mentioned by the authors, “produce valuable information for diagnostic purposes and future drug development”.

## Author contributions

All authors listed have made a substantial, direct, and intellectual contribution to the work and approved it for publication.
